# The transition experience of rural older persons with advanced cancer and their families: a grounded theory study

**DOI:** 10.1186/1472-684X-9-5

**Published:** 2010-04-26

**Authors:** Wendy D Duggleby, Kelly L Penz, Donna M Goodridge, Donna M Wilson, Beverly D Leipert, Patricia H Berry, Sylvia R Keall, Christopher J Justice

**Affiliations:** 1 Faculty of Nursing, University of Alberta, 3rd floor Clinical Sciences Building, Edmonton Alberta, T6G 2G3, Canada; 2 Nursing Division, Saskatchewan Institute of Applied Science and Technology, 4500 Wascana Parkway, Regina Saskatchewan, S4P 3A3, Canada; 3 College of Nursing, University of Saskatchewan, Health Sciences Building, 107 Wiggins Road, Saskatoon, Saskatchewan, S7N 5E5, Canada; 4 School of Nursing, University of Western Ontario, Health Sciences Addition, London, Ontario, N6A 5C1, Canada; 5 Hartford Center of Geriatric Nursing Excellence, College of Nursing, University of Utah, 10 South 2000 East Front, Salt Lake City, 84112-5880, USA; 6 Five Hills Health Region, 1000 Albert Street, Moose Jaw, Saskatchewan, S6H 2Y2, Canada; 7 Department of Anthropology University of Victoria, 3800 Finnerty Road Victoria British Columbia V8W 3P5, Canada

## Abstract

**Background:**

Transitions often occur suddenly and can be traumatic to both patients with advanced disease and their families. The purpose of this study was to explore the transition experience of older rural persons with advanced cancer and their families from the perspective of palliative home care patients, bereaved family caregivers, and health care professionals. The specific aims were to: (1) *describe the experience of significant transitions experienced by older rural persons who were receiving palliative home care and their families *and (2) *develop a substantive theory of transitions in this population*.

**Methods:**

Using a grounded theory approach, 27 open-ended individual audio-taped interviews were conducted with six older rural persons with advanced cancer and 10 bereaved family caregivers. Four focus group interviews were conducted with 12 palliative care health care professionals. All interviews were transcribed verbatim, coded, and analyzed using Charmaz's constructivist grounded theory approach.

**Results:**

Within a rural context of isolation, lack of information and limited accessibility to services, and values of individuality and community connectedness, older rural palliative patients and their families experienced multiple complex transitions in environment, roles/relationships, activities of daily living, and physical and mental health. Transitions disrupted the lives of palliative patients and their caregivers, resulting in distress and uncertainty. Rural palliative patients and their families adapted to transitions through the processes of "Navigating Unknown Waters". This tentative theory includes processes of coming to terms with their situation, connecting, and redefining normal. Timely communication, provision of information and support networks facilitated the processes.

**Conclusion:**

The emerging theory provides a foundation for future research. Significant transitions identified in this study may serve as a focus for improving delivery of palliative and end of life care in rural areas. Improved understanding of the transitions experienced by advanced cancer palliative care patients and their families, as well as the psychological processes involved in adapting to the transitions, will help health care providers address the unique needs of this vulnerable population.

## Background

Transitions are ongoing processes characterized by change for an individual [1] during which a new situation or circumstance is incorporated into their lives [2]. Individuals receiving palliative care may experience multiple transitions such as changes in treatment, symptoms, functional status and quality of life [3]. These transitions often occur suddenly and concurrently, and can be confusing and traumatic to both persons with advanced disease and their families [4]. Although palliative patients experience multiple transitions, research on this topic has generally focused on two areas: a) the transition from cure to receiving palliative care [5,6] and b) health care system transitions [7,8]. These studies have not explored the experience of transitions of older rural adults and their families once they are receiving palliative care.

The majority of persons with advanced cancer in Canada are over the age of 65 [9] and concurrently with experiencing transitions associated with advanced cancer they are also dealing with normal changes associated with aging. Normal changes with aging influence the presentation of symptoms, response to treatments and the care needs of older adults receiving palliative care [10]. Thus their transitions and how they deal with their transitions may differ from that of other age groups. As well, **r**ural residents are at higher risk for ill health and unmet health care needs as a result of living far from highly specialized and centralized health care services [11,12]. Furthermore, many additional social and community services are often found only in distant larger communities [13,14]. Living in rural/remote areas presents additional challenges for dying persons and their families, such as reduced and difficult access to needed services [15-18].

Very little research has been conducted on the experience of transitions after a person has been admitted to palliative care services. One qualitative study was found that examined transitions once a person was receiving palliative care. In this study health care professionals, family members and patients with advanced cancer and chronic obstructive lung disease (COPD) were interviewed. These participants described multiple transitions in treatment as well as symptoms, quality of life, and functional status [3]. However, this study did not explore how older rural palliative patients and their families adapt to transitions. Thus, only limited knowledge is available regarding both the experience of significant transitions once an older rural person becomes palliative and how they and their caregivers adapt to the transitions. Developing an understanding of transitions and the psychological processes involved in adapting to them is essential to addressing the unique needs of this vulnerable population.

This study was conducted to add to our knowledge regarding how older rural adults with advanced cancer and their caregivers experience transitions, and more specifically, how they adapt to them. The purpose of this study was to explore the transition experience of older rural persons with advanced cancer and their families from the perspective of palliative home care patients, bereaved family caregivers, and health care professionals. The specific objectives were to: (1) *describe the experience and psychosocial processes of significant transitions experienced by older rural persons who were receiving palliative home care and their families *and (2) *develop a substantive theory of transitions in this population*.

## Method

Transitions of older rural persons receiving palliative home care and their family members comprise a complex yet important process within a limited area of research. For these reasons Charmaz's [19] constructivist grounded theory approach was used. The study was approved by the University of Saskatchewan Behavioral Ethics Review Board (BEH-07-68) and was conducted between September 2007 and October 2008.

### Sample and Setting

Multiples perspectives of the transition experience were sought to gain a more comprehensive picture of the transition experience. Purposeful theoretical sampling was used to enroll 28 study participants from three groups: 1) rural older rural palliative care cancer patients (n = 6), 2) bereaved family caregivers (n = 10), and 3) palliative health care professionals (n = 12) from three health regions in Saskatchewan, Canada. The palliative care coordinators from the three regions identified potential participants based on the following criteria:

1. *Patients*: a) 60 years of age and older; b) diagnosed with advanced cancer; c) self-reported to live in a rural area; and d) able to participate as determined by the palliative care coordinator.

2. *Bereaved Family Caregiver*: a) age 18 years or older; b) experienced the death of an older (60 years and older) family member who received palliative care 2 to 12 months previous; c) self-reported to live in a rural area; and d) had direct contact with the decedent at least once per week in the last month of the decedent's life.

3. *Palliative Health Care Professionals: *a) age 18 years or older; and b) providing direct care to older palliative care patients in rural settings.

Participants were recruited for individual interviews or focus groups until saturation was reached (data collected was determined to be rich and sufficient, and no new properties of the categories or theoretical insights were being gained) [19]. Names and telephone numbers of interested study participants were provided to the research assistant by palliative care coordinators. Health care professionals and bereaved family caregivers who expressed interest in the study by phone were then mailed a study package. These participants completed consent forms and demographic forms prior to beginning the telephone interviews. Potential palliative care patient participants were interviewed in their homes at a time that was convenient for them after obtaining written informed consent. The palliative patients were interviewed face-to-face to reduce the study burden by allowing a more comfortable position than talking on the phone.

### Data Collection

Data collection for all participants included a demographic form and open-ended audio-taped interviews using an interview guide. The interview guide questions focused on: a) the most significant or biggest transitions experienced by palliative care patients and their family members, b) when transitions occur, c) how they deal with transitions, and d) what helped or hindered the transitions. Where possible, participants were interviewed twice to provide an opportunity to add to and clarify what was said in the first interview. The interview guide was adapted accordingly for the second follow-up interviews based on the responses and ongoing data analysis. Specific data collection methods for each participant group were as follows:

1) *Patients: *A total of seven in-depth face-to-face open-ended audio-taped interviews were conducted with six palliative patients lasting 30-60 minutes. Only one of the six patients was interviewed twice: three participants died shortly after the first interview and two were too ill to be interviewed a second time.

2) *Bereaved Family Caregivers*: Twenty open-ended, in-depth audio-taped telephone interviews were conducted with 10 bereaved family caregivers lasting approximately 50-70 minutes. All caregivers were interviewed twice.

3) *Palliative Health Care Professionals: *Four telephone focus group interviews (4-6 participants per group) were completed with 11 female palliative RNs and 1 female social worker. Group membership was determined based on the time the focus groups were able to meet. Each group was interviewed twice. Focus group interviews lasted 50-70 minutes.

### Data Analysis

Data analysis occurred as data was collected. All audio-taped group and individual interviews were transcribed verbatim. Transcripts were checked for accuracy then entered into N6 software for data management. Data were analyzed during data collection using Charmaz's [19] grounded theory approach which includes initial, focused, and theoretical coding. During initial coding, data were analyzed line by line to form categories, search for and identify processes, meanings, actions, change, and consequences. The most significant or frequent initial categories were identified during focused coding. Data were then compared to data, categories to categories, and incident to incident, to develop the properties of the focused codes. Using theoretical coding, the relationships between the categories and concepts were identified, and the focused codes integrated and organized into an emerging theory of transitions. Initially, each group's data were analyzed separately by three different graduate students and reviewed by the research team. As the overall concepts were similar in all three groups all data was integrated into the emerging theory.

To ensure emerging theory had fit, relevance, and modifiability, trustworthiness of the data was sought using the criteria of credibility, originality, resonance and usefulness [19]. Credibility and originality of the data were obtained by gathering rich, in-depth data from interviews, field notes, and memos, and by transcribing verbatim and coding line by line using the participants' words as much as possible. The results of the interviews were confirmed for resonance and usefulness with the participants in second interviews whenever possible.

## Results

### Sample

A total of 28 individuals participated in the study: a) six (two male and four female) older rural palliative care patients (mean age 73; 7.403 SD) with advanced cancer (3 Lung Cancer, 2 Stomach Cancer and 1 Pancreatic Cancer); b) 10 bereaved family members (eight female, two male; mean age 62 years; 11.24 SD) with a mean length of time care giving of 19.7 months (20.00SD) within the first year of bereavement after providing care to a family member with advanced cancer, and c) 12 (female) palliative health care professionals (mean length of time working in palliative care 11.3 years; 7.14 SD).

### Context

The social context in which the findings were interpreted were the emerging themes of isolation, lack of information, and poor communication with health care providers, lack of accessibility to services and the value of individuality and community connectedness. For example one participant described feelings of isolation: *"...so I guess, in that way, I suppose you're isolated a bit" (*P-02). Another described lack of information and poor communication with health care providers: *"...but nobody tells me anything... even the doctors*" (P-06). Lack of accessibility to services was also described: *"...they have to go, so some of our clients have had to go outside of their community a considerable distance to where they don't know anybody*."(HP-10). Fatigue and stress were also present:

"*I was really exhausted and... I guess I had it in my head, OK, I'm supposed to do this so I will do it. There isn't anybody else to help out and my daughter can't leave her children to come and help me... so this is the way it's going to be*." (C-01)

The value of individuality and community connectedness were also described by participants: As one participant said: *"I think that's the key there too, is dealing with them as individuals and, you know, remembering that everybody is different*." (HP-12) Individuality as well as a sense of community: "*And I think, on the farms, people know more about each other than in the city. You make friends in the city, so fine, but on the farm... in our area especially, if somebody was in... in some kind of problems... neighbors were there to help... So may, maybe a more of a sense of community there" (*C-06).

### Transitions

The most significant transitions of palliative care patients and their families were described as being unexpected, sudden, and new. For example, one participant said: "*But all of a sudden the relationship of husband/wife was gone*...";(C-02) and "*because everything was just so unexpected for both of us; and everything that was new to him that was a frustration*" (C-03). The transitions resulted in a disruption of their lives resulting in anxiety, distress and uncertainty but some participants suggested transitions were also beneficial in terms of forming closer relationships with their family. As one participant said: "*Best thing about the changes?...our family got a little bit closer, they care for me more*..." (C-09).

Four overlapping themes reflected the transitions: a) environmental, b) relationships and roles, c) physical and mental health, and d) activities of daily living. Table 1 presents an overview of the transitions themes as well as examples of data from all three participant groups. Environmental transitions included a) physical transitions within the home environment during the person's illness, b) being unable to leave their homes, and c) care setting changes from home to hospital and long term care facilities. Significant changes occurred in roles/relationships. For example, instead of caring for others or being able to work and provide for others, patients were now dependent on others. Changes in roles and relationships for palliative patients also occurred with changes in physical and mental health. Disease progression and medications resulted in changes to palliative patients' physical and mental health such as fatigue and other symptoms as well as changes in their mood and ability to think clearly. These changes reduced the palliative patients' ability to care for themselves, thus altering their activities of daily living.

**Table 1:** Themes of Transitions and Data Examples

Themes	Data Examples
**Environmental Changes**	"She was an outdoor person, so naturally it was a big change, but she did accept it and of course her condition was making her realize that she... couldn't be outdoors." (Family Caregiver) (C-03)
	"It just seems like their whole house is turned up topsy turvy as you're bringing in all these supplies..." (Health Care Professional) (HCP-02) "...being in the hospital, because I was in the hospital... and I can't tell you how astounded I was" (Palliative Patient) (P-03)

**Relationships and Roles**	"I really just felt like a nurse myself... That, that was how I felt. I mean, I used to work as a nurses aide in a nursing home... and I do have some... you know, knowledge... of that kind of thing... But all of a sudden the relationship of husband/wife was gone and it was nurse and patient for me... That was our, a big change." (Family Caregiver) (C-06)
	"...in a rural setting, people have very specific rules if they're a farmer, whatever their profession... they have to face the inability to protect or care for their family" (Health Care Professional) (HP-04) "You are always used to helping out, and now they've got to help you...." (Palliative Patient) (P-05)

**Physical and Mental Health**	"And I think like the rapid change in her condition, the weight loss, the jaundice, the diabetes, um, her incontinence, you know... that really... I, I think that really bothered her... Well it was her jaundice... number one was very, very acute. Um, she just wasn't eating - couldn't hold anything down. Um, she began getting more blood in her stools." (Family Caregiver) (C-07)
	" When we first come on a case, I have found a lot of times we're dealing with pain control, a control of symptoms is one of the biggest things" (Health Care Professional) (HP-08) "...as when something gets to a point and it's really, I'll sound like an idiot, it's like all of a sudden you have this brain problem...." (Palliative Patient) (P-05)

**Activities of Daily Living**	"Um...you know, she wanted to be the boss and that's all there was to it. ... But, you know, we did the general housekeeping things, we looked after her yard and her garden, did some shopping for her, and we were just there to spend time... Just to be with her." (Family Caregiver) (C-10)
	"I think that one of the biggest changes or transitions that the palliative care patients go through is loss of independence and having to rely on someone else." (Health Care Professional) (HP-11) "...I miss doing my usual stuff. Working for the hall, doing work at home, doing charity work... I miss doing that." (Palliative Patient) (P-06)

### Factors Influencing Transitions

Factors helping palliative patients and their families adapt to transitions were timely communication, the provision of essential information, and the presence of family/community support networks. The need for timely communication about essential information was illustrated by this comment: "...*if we would have known more of what to expect, what the symptoms are...if we had known before, it would have been a lot easier"(*C-02). A supportive network of friends and community was also beneficial as illustrated by this comment about a rural community: "*One thing I'll say about it all, like being in a small community, it was help in a sense to know that we had neighbors who we could call if we were in trouble" *(C-04).

### Basic Social Process: Navigating Unknown Waters

Transitions disrupted the lives of palliative patients and their families. They experienced feeling out of control ("...*everything I guess was out of control"*), (P-02) did not know what to do or what to expect ("...*it was way beyond my knowledge...didn't know what to do"*),(C-03) and anxiety ("...*It was hard, so very very hard... panic started to set in"*) (C-05).

The emerging overall process by which older rural palliative patients and their families adapted to transitions was "Navigating Unknown Waters". The basic social process represents all the data and the name of this process was based on the words from the participants. As one participant said: "*there's too many landmines and changes happening as it is... that you're trying to navigate... unknown waters*" (C-04). This basic social process included the sub-processes of: a) coming to terms with their situation, b) connecting with others, and c) redefining normal.

#### a) "Coming to terms" with their situation

Coming to terms with their situation was the first process for palliative patients and their families in dealing with their transitions. For example one participant said: "...*you have to come to terms, you know.... with the change" *(P-03). Coming to terms was not acceptance, but an awareness, understanding, or acknowledgement of their situation. Older rural palliative patients and their families were able to come to terms with their situation by reminiscing and reframing their hope. Reminiscing (or life review) about who they once were compared to where they were now was helpful in coming to terms with their situation (e.g., "...*thinking about your life and what you've got in your life*.") (C-03). Reframing their hope was also helpful during this process: *"...and they just feel that they kind of have to hope... that's part of reframing hope to me*" (HP-07).

#### b) Connecting with others

After they were able to acknowledge their situation, the sub-process of connecting with others occurred, during which palliative patients and their families actively sought information, searched for options, and connected with trusted experts to help adapt to transitions:

'*Um, we had sought out a lot of information, so as this cancer progressed through the different stages we were somewhat prepared for what to look out for and what would be happening with mother...so it wasn't that it was unexpected or anything, it was just....the issue of dealing with it*.... *Try different things, but not everything works*" (C-04).

Family caregivers and palliative patients described the importance of information coming from whom they considered to be experts: *"And as the time went by, we realized that the palliative care nurse was so different than a registered nurse... had extra training and that was very evident every time they came to our house. ...it gave us a sense of being looked after"(C-01)*.

#### c) Redefining normal

Palliative patients and their families changed what they considered to be normal: "...*like we said, you know, redefining everything in your world" (*P-02). They defined new standards of what was "well": *"...I'm feeling well... normal... Yes, normal for being sick*" (P-04). Once they knew what was normal for their situation, then they were able to determine when they should worry and seek care: *"he was having troubles breathing, but he was okay... he didn't have pain*" (C-01).

In the process of redefining normal, maintaining their personhood--who they were--was very important. As one participant explained: "....*because when you're sick like everybody says ...I'm still me you know....I don't change*. *I deal with the changes by being me*..." (P-05).

**Figure 1:**
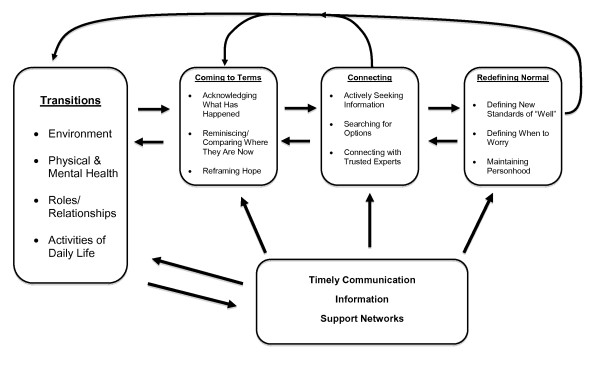
**Navigating Unknown Waters**.

The processes of the emerging theory of "Navigating Unknown Waters" were interrelated and overlapping and constantly interacting as illustrated in Figure 1. The arrows indicate inter -relationships and constant interaction of the sub-processes. The processes were overlapping, however for clarity are depicted in the figure as separate. The first process to occur was coming to terms with their situation. Once they were able to come to terms older rural palliative patients and their families were then able to connect with others and subsequently redefine what was normal within their situation. Normalizing their transitions helped them to adapt to them and reduced stress and uncertainty. Timely communication, provision of information and support networks facilitated the process of "Navigating Unknown Waters". These processes occurred within the context of multiple co-existing transitions, receiving rural palliative home care and dealing with advanced cancer.

## Discussion

The tentative theory of "Navigating Unknown Waters" is contextually situated in a rural context and end of life experiences of rural older palliative care patients and their families. The rural context of isolation, individuality, and independence is well documented in other studies of rural health [20,21]. The findings from our study raise the issues of lack of services and poor communication with health care professionals within a rural setting as well as the importance of individuality and connectedness with the community.

Within the study context, multiple transitions occurred in the areas of environment, physical and mental health, activities of daily living, and roles and relationships. As mentioned previously much of the literature on transitions in palliative care has focused on physical transitions of care settings. However the changes within the home environment to accommodate the increasing physical limitations of a person with advanced disease and the shrinking of their physical environment when they are unable to leave their home have received little attention in the research literature. These transitions within the home environment are often difficult for palliative patients and their families and require further research.

Transitions in physical and mental health as well as activities in daily living have been described in another study of transition with persons with advanced cancer and COPD [3]. However transitions in roles and relationships and the broadly defined transitions within the home environment were not described. It is unclear if participants in the above study were receiving palliative home care service, were older or were in a rural or urban setting making it difficult to determine why their study findings differed from ours. Research is needed to determine if the types of transitions experienced by older rural palliative care patients and their families are similar to those in other age groups and geographic locations.

Transitions resulted in disruption in the lives of palliative care patients and their families. This disruption was dealt with through the process of "Navigating Unknown Waters" which included its sub-processes of coming to terms with the situation, connecting with others, and redefining normal. These processes helped reduce the distress and uncertainty of transitions. The sub-process of " coming to terms" with their situation has also been described in a study of persons with advanced disease and their family caregivers as a way of negotiating and coping with a terminal prognosis [22]. Similar to the findings of our study palliative patients coped with a terminal prognosis by managing awareness of their situation through reframing their hope. The authors did not however report the use of reminiscing to help manage awareness. Older palliative patients have described life review and reminiscing as ways to transform hope [23]. As our study was focused on transitions of older palliative patients and their families, life review and reminiscing may be unique to older palliative patients.

The importance of timely communication of information has been described by participants in studies of the transition from a curative focus into palliative care [6,5]. As well, actively seeking information was a way of fostering hope for older palliative patients and their families [23,24]. However, our findings of actively seeking information, searching for options and connecting with trusted experts as components of the sub-process of connecting with others has not been previously described in the literature. The sub-process of connecting with others may be unique to the rural context as the value of community connectedness described in our study has also been identified as a feature of rural communities [20,21]. Further research is needed to determine if this is the case.

The sub-process of redefining normal occurred through changing definitions of what was "well" and when to worry as well as maintaining personhood. Our data suggested that redefinition of "normal" was not based on what others exhibit, as has been suggested in other transition theories [25,26], but involved coming to terms with their situation and connecting with others. As well rural people may not redefine what is normal based on what others exhibits because there maybe few others in a sparsely populated rural area with the same diagnosis or symptoms and have limited opportunities to connect with others with similar issues.

The emerging theory of "Navigating Unknown Waters" is conceptually different from existing transitions theories in that our study highlights: a) the importance of the relational aspect of dealing with transitions and b) the importance of maintaining personhood throughout the process. Existing transition theories [25-27] do not appear to include the reciprocal and interactive dimensions of transitions [28]. The nature of the transitions and responses to transitions of palliative care patients may not have the same outcomes and adaptations seen in other populations [6]. Our study findings suggest the relational and interactive dimensions are important in the transition experience of older rural persons with advanced cancer and their families. For example, connecting with others enabled older rural palliative patients and their families to deal with uncertainty and distress of significant transitions. Furthermore, the process of redefining normal did not appear in our study to involve a redefinition of self as has been described in the chronic illness literature [2,29] or other studies of transitions [6]. The importance of maintaining who they were as they redefined normal was unique to our study of transitions. Maintaining personhood, although not previously described in the transition literature, has been described as an important process for palliative care patients [30,31].

### Limitations

Several factors influenced this study, including the incorporation of multiple perspectives--patients, bereaved family caregivers, and health care professionals--in identifying the emerging theory. Health care professionals have been found to identify different transitions than patients and families in a study of advanced cancer and COPD [3]. However, the themes identified in our study were found in the data from all participants. As transitions were not studied over a long period of time, multiple perspectives were needed to capture the transitions experience throughout the end of life experience, and appeared to facilitate a deeper understanding of transitions.

Other limitations include the characteristics of study participants such as geographic location and cancer sites. Further research is also needed to validate the theory in locations other than rural western Canada, as both location and access to resources may impact how older palliative advanced cancer patients and their families adapt to transitions. The study participants had a range of primary cancer sites. Palliative populations, such as those with other primary cancer sites and non-cancer diagnoses, may have different transition experiences. For example, the transition experience of persons with advanced cancer can differ from persons with COPD [3]. Further research is needed to determine if the processes identified in this study are unique to individuals with advanced cancer and their caregivers or are shared with others. The developed theory is tentative and further research is needed to explore the relationships between the processes and whether the order in which the processes occur is of importance. The findings of this study and possible transferability of the findings need to be interpreted considering the factors influencing this study and the social context of the participants.

## Conclusion

The findings of our study address a gap in the literature on the nature and experience of transitions of older palliative patients and their family caregivers within a rural context. The uncertainly, lack of control, and distress described by the study participants highlight the critical need to address the significant transitions of environment, physical and mental health, roles/relationships and activities of daily living. These transitions may serve as a focus for improving delivery of rural palliative care.

The tentative emerging theory of "Navigating Unknown Waters" appears to add to our knowledge of the transition experience of older rural palliative care patients and their families. As such it provides a foundation for future research and it may provide a useful approach in the development of intervention tools/strategies to diminish the distress experienced by both rural persons with advanced cancer and their family members as result of transitions. For example, rural palliative health care professionals may choose to focus on reminiscing (life review) and reframing of hope, two strategies that can be employed by dying persons to come to terms with their situation. By searching for meaning through life review, and establishing new patterns of hope, palliative patients are better able to maintain relationships with others [23]. Palliative care professionals also have the opportunity to connect with their patients as trusted experts by providing practical information on what may help/hinder the progression of the transitions and by discussing options with all individuals involved in the patients' care. Timely communication and the provision of information and support networks are all potential ways health care professionals may facilitate the processes of "Navigating Unknown Waters".

## Competing interests

The authors declare that they have no competing interests.

## Authors' contributions

All authors contributed to the development and design of the study. WD and KP were involved in the data collection and analysis. WD was responsible for the overall organizing and drafting the manuscript, with KP, DG, DW, BL, PB, SK, CJ contributing to writing a section of the paper. All authors read and approved of the final manuscript.

## Pre-publication history

The pre-publication history for this paper can be accessed here:

http://www.biomedcentral.com/1472-684X/9/5/prepub
